# Evaluation of the passage of *Lactobacillus gasseri *K7 and bifidobacteria from the stomach to intestines using a single reactor model

**DOI:** 10.1186/1471-2180-9-87

**Published:** 2009-05-08

**Authors:** Philipp Ritter, Christian Kohler, Ueli von Ah

**Affiliations:** 1 Agroscope Liebefeld-Posieux Research Station ALP, Schwarzenburgstrasse 161, CH-3003 Bern-Liebefeld, Switzerland

## Abstract

**Background:**

Probiotic bacteria are thought to play an important role in the digestive system and therefore have to survive the passage from stomach to intestines. Recently, a novel approach to simulate the passage from stomach to intestines in a single bioreactor was developed. The advantage of this automated one reactor system was the ability to test the influence of acid, bile salts and pancreatin.

*Lactobacillus gasseri *K7 is a strain isolated from infant faeces with properties making the strain interesting for cheese production. In this study, a single reactor system was used to evaluate the survival of *L. gasseri *K7 and selected bifidobacteria from our collection through the stomach-intestine passage.

**Results:**

Initial screening for acid resistance in acidified culture media showed a low tolerance of *Bifidobacterium dentium *for this condition indicating low survival in the passage. Similar results were achieved with *B. longum *subsp. *infantis *whereas *B. animalis *subsp. *lactis *had a high survival.

These initial results were confirmed in the bioreactor model of the stomach-intestine passage. *B. animalis *subsp. *lactis *had the highest survival rate (10%) attaining approximately 5 × 10^6 ^cfu ml^-1 ^compared to the other tested bifidobacteria strains which were reduced by a factor of up to 10^6^. *Lactobacillus gasseri *K7 was less resistant than *B. animalis *subsp. *lactis *but survived at cell concentrations approximately 1000 times higher than other bifidobacteria.

**Conclusion:**

In this study, we were able to show that *L. gasseri *K7 had a high survival rate in the stomach-intestine passage. By comparing the results with a previous study in piglets we could confirm the reliability of our simulation. Of the tested bifidobacteria strains, only *B. animalis *subsp. *lactis *showed acceptable survival for a successful passage in the simulation system.

## Background

Probiotics, especially lactic acid bacteria have beneficial effects on consumers health as suggested in 1907 [1]. It was believed that bacteria mainly controlled infections caused by enteric pathogens and regulated toxoaemia, thereby improving health and influencing mortality. Meanwhile it has been known that some of the positive effects on consumers health are the improvement in the microflora balance in the gut, the stimulation of the immune system, and aiding the organism to fight pathogenic microorganisms [2]. A large part of interest was concentrated on the use of strains of the genera *Lactobacillus *and *Bifidobacterium*, even if there are also other bacteria with probiotic effects, e.g. some propionibacteria.

The above mentioned properties are also the basis for a microorganism to be labelled probiotic. There are different definitions worldwide but they are similar in content. One of the criteria for a probiotic strain is its resistance to acidity and gastric solutions in the human gastrointestinal tract [3]. It is therefore important, to evaluate the resistance of a potential probiotic strain to the acidic and gastric environment in the intestine.

Because of high costs and ethical as well as safety regulations for clinical studies, screening survival is easier to simulate *in vitro*. A simple test is to incubate the bacterial cells in acidic or bile salt solutions for a defined period and count the number of surviving cells. In a further step, the simulation is carried out in agitated flasks, combining acidity and gastric solutions followed by an estimation of surviving cells over the entire simulation. This is a more realistic replication of the conditions in the intestine [4]. Another system, the Simulator of the Human Intestinal Microbial Ecosystem (SHIME), consists of 5 to 6 serially connected pH controlled bioreactors [5-7]. The setup is quite complex and demands absolute anaerobic conditions. Furthermore, the absorption of metabolites and water is not simulated. This was overcome by using dialysis membranes as described by Marteau *et al*. [8].

Recently, a new system using a single bioreactor was developed to study the stomach-intestine passage [9]. The system allowed the pH to be altered inside a single reactor and was adapted to the retention times in the different regions of the stomach-intestine passage.

*Lactobacillus gasseri *K7 was recently isolated from infant faeces [10]. It produces a bacteriocin which is active against *Clostridium *sp. and their spores. *L. gasseri *belongs to the so called "acidophilus"-group and several independent studies identified these strains as inhabitants of the skin and intestine [11-13]. In previous experiments, it has already been shown *in vitro *that *L. gasseri *K7 survived in an acidic environment and with 0.3% bile salts [10]. These findings make the strain interesting as a possible probiotic.

In this study, a single bioreactor system based on the work of Sumeri *et al*. [9] was used to evaluate the survival of *Lactobacillus gasseri *K7 and eight *Bifidobacterium *strains from our collection. We were able to compare the results for *L. gasseri *K7 with a study performed in piglets [14] which allowed the assessment of a correlation between the *in-vitro *study with results from *in-vivo *experiments.

The retention times and pH used in this study were based on data from the literature. Several methods exist for measuring the pH in the intestine [15]. Table 1 shows the pH values in the different parts of the intestine as measured by the Heidelberg capsule [16,17]. Retention times can be calculated either by using marker substances (chemical) or by radio telemetry capsules such as the *Heidelberg *capsule [18]. However, capsules usually have longer retention times than chemical markers. Table 2 lists some of the retention times found in the literature [4,5,19-24].

**Table 1:** pH values in the human intestinal tract, measured with the *Heidelberg *capsule.

	Stomach	Duodenum	Jejunum	Ileum	
			proximal	medial	Distal
**pH**	1.4**	6.22*	6.4**	7.1**	7.4**

* Fallingborg *et al*. 1994 [16]

** Fallingborg *et al*. 1998 [17]

**Table 2:** Retention times in the small intestine cited in literature.

Retention time	Source	Remarks
1–4 h	Huang and Adams 2004 [21]	
4.25 h	Van Den Driessche *et al*. 2000 [24]	Stomach and small intestine
4 h	Mojaverian 1996 [22]	
6 h	Picot and Lacroix 2004 [4]	Selected maximum time of the simulation
7.5 h	Fallingborg *et al*. 1990 [20]	Children
8 h	Fallingborg *et al*. 1989 [19]	
8 h	Alander *et al*. 1998 [5]	Simulation in the SHIME Reactor
6–10 h	Thews *et al*. 1991 [23]	

**Figure 1:**
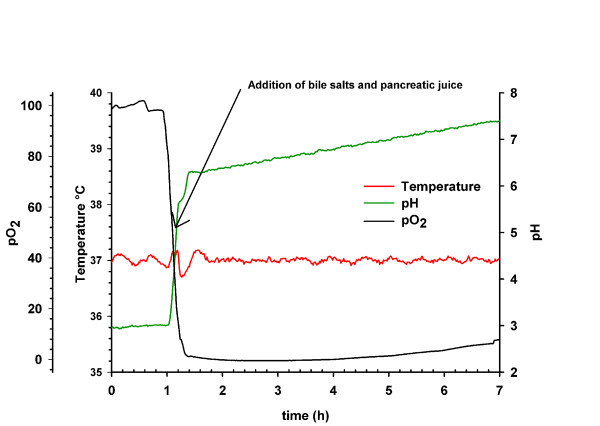
**Parameters of the stomach-intestinal passage simulation over 7 h**.

Based on the data found in the literature and the work by Sumeri *et al*. [9] the fermentation process was set up as described in Material and Methods and is shown in Figure 1.

## Results

### Acid resistance screening

**Figure 2:**
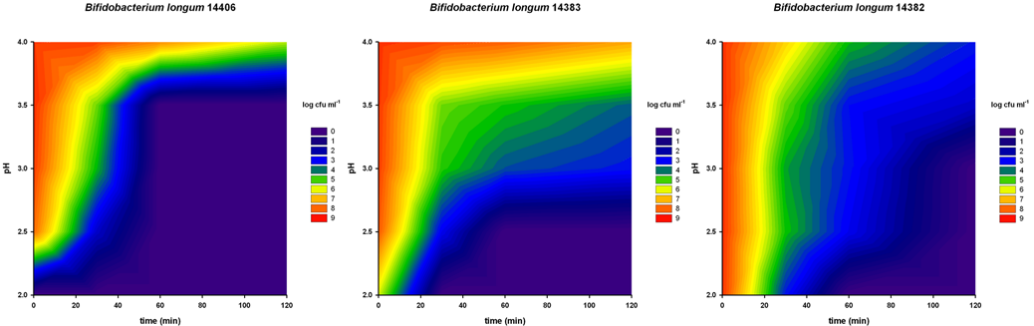
**Acid resistance of three *Bifidobacterium longum *strains**. X-axis: time (min); Y-axis: pH; log cfu are shown in colour (scale on the right of the graphs). Numbers in the bacterial names are the strain numbers in the FAM-database of ALP.

**Figure 3:**
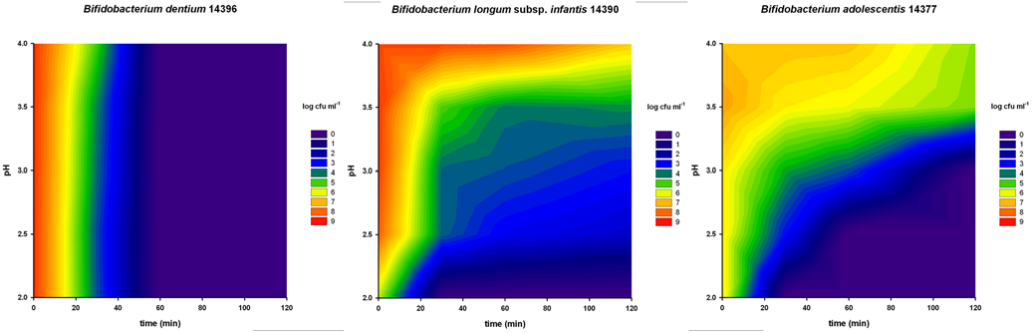
**Acid resistance of *Bifidobacterium dentium*, *B. longum subsp. infantis *and *B. adolescentis***. X-axis: time (min); Y-axis: pH; log cfu are shown in colour (scale on the right of the graphs). Numbers in the bacterial names are the strain numbers in the FAM-database of ALP.

**Figure 4:**
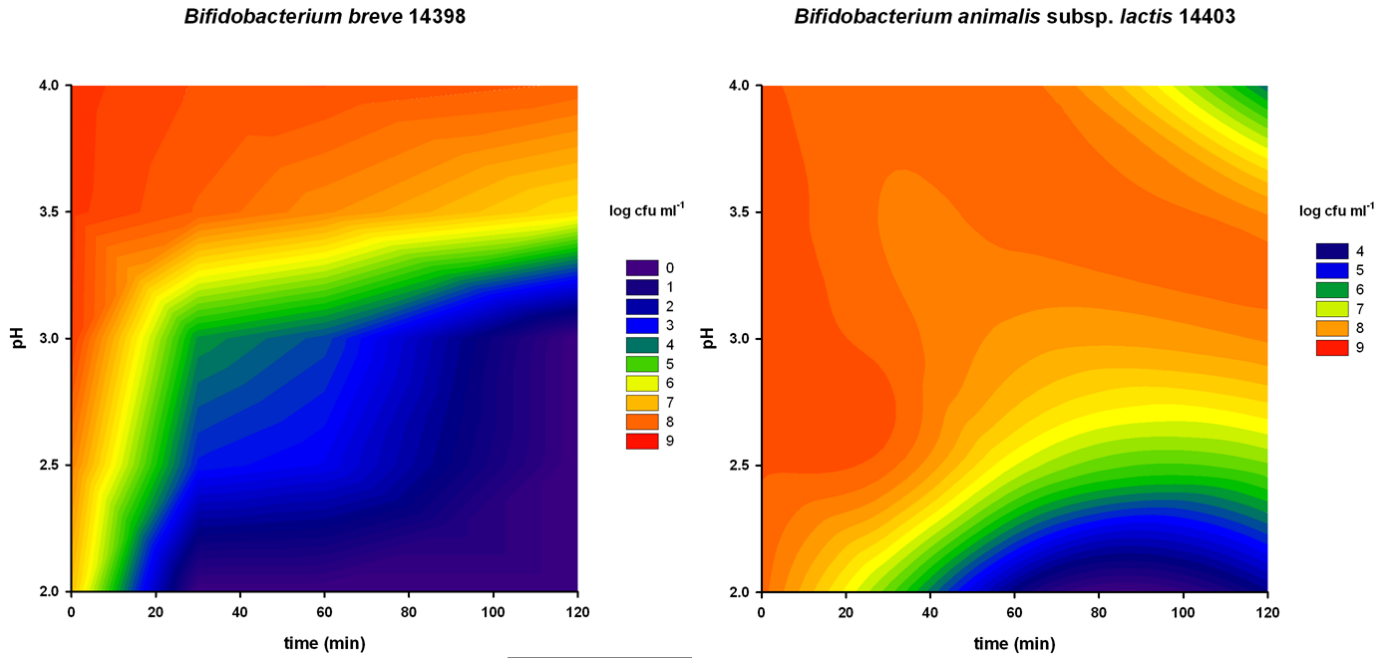
**Acid resistance of *Bifidobacterium breve *and *B. animalis *subsp. *lactis***. X-axis: time (min); Y-axis: pH; log cfu are shown in colour (scale on the right of the graphs). Numbers in the bacterial names are the strain numbers in the FAM-database of ALP.

The aim of an initial series of tests was to obtain an overview of the acid resistance of eight bifidobacteria strains. Figures 2, 3 and 4 show the survival of these strains using contour plots made with Sigmaplot. *Bifidobacterium dentium *(Figure 3) showed the least acid resistance. Between pH 4.0 and pH 2.0 there was no difference in survival and the concentration of cells dropped by more than 7 log within 40 minutes. *Bifidobacterium animalis *subsp. *lactis *was more resistant up to 40 min at pH 2.0, but then decreased by about 3 log when incubated for 120 minutes (Figure 4). At a pH between 2.5 and 3.0 the decrease was less than 1 log after 120 minutes.

All the other tested *Bifidobacterium *strains (*B. longum, B. breve, B. longum *subsp. *infantis and B. adolescentis*) showed a similar but different pattern from *B. animalis *subsp. *lactis *(Figures 2, 3 and 4). They had a short survival time below pH 2.5 and survived in higher numbers above pH 3.5.

**Figure 5:**
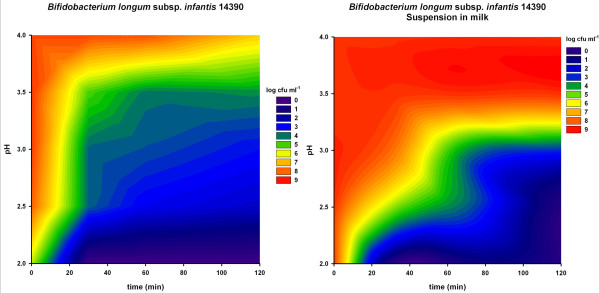
**Comparison of acid resistance of *Bifidobacterium longum *subsp. *infantis *14390 suspended in NaCl or skim milk**. Left: Bifidobacteria resuspended in NaCl, right: Bifidobacteria resuspended in milk. X-axis: time (min); Y-axis: pH; log cfu are shown in colour (scale on the right of the graphs). Numbers in the bacterial names are the strain numbers in the FAM-database of ALP.

With the aim of developing a method to simulate the GI in the bioreactor, a further test was done with one strain. To observe the influence of a food matrix, concentrated *B. longum *subsp. *infantis *was resuspended in skim milk before inoculating into acidic solutions. As shown in the right-hand column of Figure 5, milk had a direct effect on the survival of the strain. Between pH 3.0 and 3.5 the bacteria survived for 120 min with a reduction of log 2. Below pH 3.0 the survival rate decreased to about log 5. The decrease in survival below pH 3.0 was rapid but regular over time. At pH 3.5 and above, the strain was resistant for at least 120 minutes.

The left-hand column of Figure 5 shows the same strain without added skim milk. At a pH above 3.5, there was no influence on the survival of the bacteria. However, below pH 3.5 the survival decreased depending on the duration of incubation. Between pH 3.0 and 3.5 the strain had already decreased by about log 5. After 30 min incubation, there was almost a linear decrease in survival with decreasing pH from 3.0 to 2.5.

### Simulation in the bioreactor

Most systems described in the literature consist of several reaction vessels, e.g. the SHIME [6]. Other studies used immobilized cells with three reactors [25] or a dialysis system [8]. Based on the work of Sumeri *et al*. [9] and the collected data of the conditions in the intestinal passage we were able to limit the simulation to one vessel. Together with the data from the acid resistance screening, the selection of a possible starting pH and broth composition in the simulator could be chosen. The resulting simulation parameters are shown in Figure 1 and described in the Material and Methods section. During the experimental stage of this study, Sumeri *et al*. [9] developed a similar system to evaluate *Lactobacillus *sp. in a stomach-intestine passage simulation.

**Figure 6:**
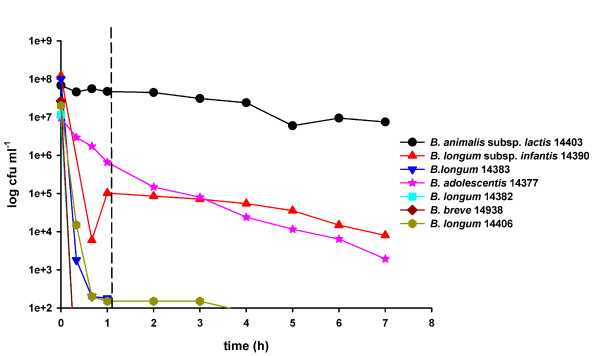
**Development of 7 *Bifidobacterium *strains during stomach-intestinal passage simulation for 7 h**. Dashed line shows the time of addition of bile salts and pancreatic juice. Numbers in the bacterial names are the strain numbers in the FAM-database of ALP.

The software package "Lucullus" was an excellent tool to control the pH and the process according to the developed simulation. Selecting the medium in the bioreactor was simplified by choosing the corresponding growth medium for the strains, supplemented with skim milk, functioning as a simulated food matrix. Afterwards, it was acidified to the starting pH and supplemented with enzyme solutions as described in Materials and Methods. The simulations were carried out serially, one per day. The results are shown in Figure 6. The strains used for the simulation are listed in table 3 (only *Bifidobacterium dentium *was excluded) and were standardized to an OD_650 _of 1.5 prior to inoculation.

**Table 3:** Strains tested in the simulation.

Name	Identification number of ALP strain collection
*Bifidobacterium adolescentis*	FAM-14377
*Bifidobacterium breve*	FAM-14398
*Bifidobacterium longum *subsp. *infantis*	FAM-14390
*Bifidobacterium animalis *subsp. *Lactis*	FAM-14403
*Bifidobacterium dentium*	FAM-14396
*Bifidobacterium longum*	FAM-14382, -14383, -14406
*Lactobacillus gasseri *K7	FAM-14459

*Bifidobacterium adolescentis *was inoculated as described above at an initial concentration of 10^7 ^cfu ml^-1 ^and decreased almost linearly to below 10^4 ^cfu ml^-1 ^after 5 hours. *B. breve *and *B. longum *strains had an initial concentration between 10^7 ^and 10^8 ^cfu ml^-1 ^and diminished to below 10^2 ^cfu ml^-1 ^within the first 30 minutes. *B. animalis *subsp. *lactis *14403 survived to approximately 15% of the initial average cfu of 5 × 10^8 ^cfu ml^-1^. There was a rapid decrease in survival of *B. longum *subsp. *infantis *over the first 30 min. Afterwards the survival decreased only slowly from 10^5 ^to 10^4 ^cfu ml^-1^.

**Figure 7:**
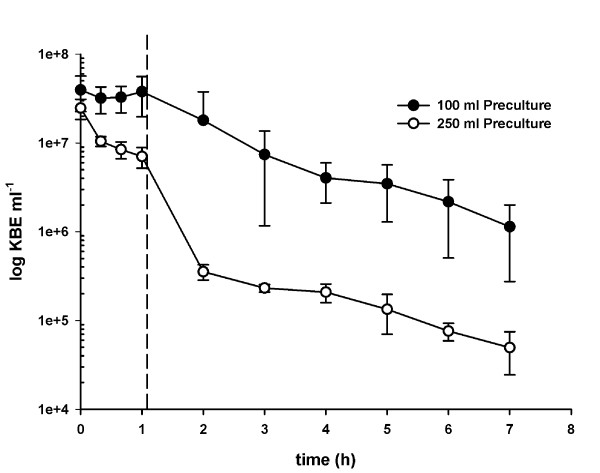
**Comparison of the influence of 100 ml pre-culture of *Lactobacillus gasseri *K7 with 250 ml pre-culture**. The pre-culture was harvested by centrifugation and resuspended in physiological sodium chloride solution to achieve an OD_600 _of 1.5. The stomach-intestinal passage simulation was incubated using the adjusted solution and incubated for 7 h. The dashed line shows the addition of bile salts and pancreatic juice. Curves are the mean of duplicate experiments.

In a later phase, *Lactobacillus gasseri *K7 was included in the study since several projects were running at this time at our institute with this strain. *Lactobacillus gasseri *K7 was inoculated at 2.2 × 10^7 ^cfu ml^-1 ^and after 7 h simulation a concentration of 10^5 ^cfu ml^-1 ^living cells was still present in the culture media (Figure 7, curve for 250 ml pre-culture). The highest reduction in survival was within the first 2 hours and began immediately after the addition of gastric juice and bile salts. Within this time, there was a reduction of living cells by log 2. During the rest of the simulation time, there was only a log 1 reduction of living cells.

The preparation of the inoculum of *L. gasseri *K7 in a 100 ml culture volume was also evaluated. The results of the experiments are shown in Figure 7. With 250 ml culture the decrease in living cells was about log 2 whereas the decrease with a 100 ml culture was only log 1 over the whole incubation time. However, 2 h after addition of bile salts and pancreatic juice, the decrease in cell counts was similar for both volumes.

## Discussion

When harvesting a culture after a given incubation time, the growth phase of each bacterial strain can be different since all have different growth dynamics. In order to obtain cells at approximately the same growth phase, preliminary experiments were performed (data not shown). An incubation time of 15 h for the pre-culture was suitable for all tested strains except *Bifidobacterium longum *subsp. *infantis *which needed to be incubated for only 12 h.

The acid tolerance screening (Figures 2, 3 and 4) was performed to evaluate the effect of pH independently of other conditions. *Bifidobacterium dentium *was highly sensitive to acid and therefore would possibly not survive the passage through the stomach. The strain was therefore not included in the simulation experiments. The *B. longum *strains (Figure 2) did not yield much better results than *B. dentium *(Figure 3). However, close to pH 4 they were more resistant than *B. dentium*.

*B. longum *subsp. *infantis *is one of the first species to populate the human intestine shortly after birth [26]. Based on the experiments in this study, however, the tested *B. longum *subsp. *infantis *strain would only be able to pass the infant stomach in high numbers if the transition time in the acidic stomach was very short. The survival of the selected strain in the tested environment was too low for successful passage in high numbers. When the strain was resuspended in skim milk, survival increased (Figure 5). This could be an indication that human milk helps *B. longum *subsp. *infantis *strains to pass the stomach-intestine passage with at a higher survival rate.

The protective effects of milk proteins in the digestive system have already been described in the literature [27]. Protection with milk proteins has also been shown in this study (Figure 5). With the appropriate matrix or even a carrier, probiotic bacteria could safely pass through the stomach to the intestines to reach their site of action.

*B. adolescentis *strains that populate the human intestine at a later age, had slightly higher resistance than *B. longum *subsp. *infantis *which may explain the reduction of the latter during the progress of the human infant to adulthood [26].

The most interesting strain was *B. animalis *subsp. *lactis*, which was the least sensitive strain in our study. This pH-resistant strain has a great potential for use in foods as a probiotic supplement since a higher number of bacterial cells would survive the passage. However, to use this strain as probiotic, more studies have to be performed in order to achieve the probiotic status according to the definition of Klaenhammer [3].

In our study, the ingestion of a food matrix was simulated in an initial environment of acidified milk and growth medium. The added simulated gastric solution and oxygen during the stomach phase increased the stress. During the simulated passage to the small intestine the oxygen was replaced by nitrogen and the medium was neutralized to pH 6.3. The addition of the pancreatic solution and bile salts completed the passage into the small intestine. This *in-vitro *system did not take into account that in *in vivo *digestion, enzymes are activated and inactivated and other substances, e.g. bile salts are reabsorbed. Sumeri *et al*. [9] found a partial solution to bypass this problem. They diluted the content of the reactor with a specially designed dilution medium. Another possibility would be to precipitate the bile salts at the end of simulation of the small intestine to imitate the enterohepatic circuit. This could be performed with calcium ions [28-30]. Removing the bile salts would better simulate the environment of the colon and might even allow bifidobacteria to proliferate.

In our study, the remaining bile salts and pancreatic juice in the simulation led to an additional stress on bacteria which probably altered the true characteristics of the strains *in vivo*.

The starting cfu in the simulation varied within one log cfu even though the adjustment of OD_650 _of the inoculum was previously tested with the *Bifidobacterium animalis *subsp. *lactis *and *Bifidobacterium longum *subsp. *infantis *strains. The bifidobacteria used in this study showed a tendency to form clusters that may result in reduced cfu (visual observations, data not shown). In another study, the formation of clusters could be related to decreasing pH during growth [31]. These clusters are usually counted as one colony on a plate.

Figure 6 shows the results of the stomach-intestine passage simulation over 7 h of seven tested *Bifidobacterium *strains. The concentration of living cells of bifidobacteria decreased immediately after incubation due to the low pH (pH 3.0). However, *B. animalis *subsp. *lactis *remained stable. This confirmed the results of previous experiments discussed above (Figure 4). This resistance could be extended to bile salts and pancreatic juice although the cell counts of *B. animalis *subsp. *lactis *decreased by about 85% of the initial value (Figure 6). Compared to the other strains used in this study, however, this decrease was almost negligible.

All *B. longum *and *B. breve *strains died very rapidly at the beginning of the simulation and were below the detection limit of the plating method within a few hours (Figure 6) which was to be expected from the results of the screening experiment above (Figures 2 and 4).

On the other hand, *B. longum *subsp. *infantis *14390 decreased rapidly at the beginning of simulation but after the addition of pancreatic juice and bile salts and a change to an anaerobic environment, the reduction rate decreased. Our study suggests that this strain is well adapted to the conditions in the intestine but needs to be ingested in high numbers to survive the conditions in the stomach (oxygen, low pH). As mentioned above, *B. longum *subsp. *infantis *strains belong to the first group of bacteria populating the intestine of infants [26].

In contrast to *B. longum *subsp. *infantis*, *B. adolescentis *decreased almost linearly during the 7 h simulation. There was no detectable interruption when the conditions in the fermenter changed. Based on the experiments for the acid tolerance screening, this result was unexpected.

However, this might be related to the testing conditions where the bile salt and gastric juice concentrations remained at the initial level and were not diluted as they would be *in vivo*. In a future experiment, it should be evaluated whether the dilution method developed by Sumeri *et al*. [9] would stabilize the cell counts of *B. adolescentis *during the 6 h simulation period in the intestine.

In our study, we also evaluated the stomach-intestine passage of *Lactobacillus gasseri *K7. The strain has already been evaluated for survival *in vivo *in piglets [14]. Therefore, it was possible to compare our *in-vitro *results with data from *in vivo *experiments.

Bogovic *et al*. [14] fed piglets over a period of 14 days with 5*10^10 ^cfu day^-1 ^of *L. gasseri *K7. This resulted in approx. 7*10^4 ^cfu g^-1 ^in the faeces during the feeding period. It has to be taken into account that the concentration of bacteria was diluted before it finally arrived at the stomach-intestine passage. In a rough approximation, we estimated that about 1% arrived at the passage. This allowed us to compare the results of this piglet study with the end of our simulation.

As shown in Figure 5, *L. gasseri *K7 had a cell concentration of approximately 5*10^4 ^cfu ml^-1 ^after the 7 h simulation period (with a pre-culture of 250 ml) which is similar to the concentration in the faeces of the piglets. This suggests that the simulation model used in this study could be a helpful tool to estimate the effects of the passage in an *in-vitro *model prior using expensive *in vivo *models. The model could be further optimized by diluting the bile salts and pancreatic juice as described by Sumeri *et al*. [9]. To simulate the activation and deactivation of enzymes a suitable method has still to be found.

When only 100 ml medium was used for the inoculum of *L. gasseri *K7, the culture survived the simulation better (Figure 7). Both volumes had a similar initial cell count. Both volumes were inoculated by 1 ml. Therefore, the culture with 250 ml volume was in an earlier stage of growth than the 100 ml culture. These results were an indication of the growth phase dependency of the culture for during stress.

## Conclusion

In this study, we were able to show that the system to simulate the stomach-intestine passage developed by Sumeri *et al*. [9] was suitable for the assessment of survival of 8 *Bifidobacterium *strains and *Lactobacillus gasseri *K7 even though we did not simulate the removal of gastric juice and bile salts. For *L. gasseri *K7 we were able to compare the results with an *in-vivo *study on piglets and obtained similar results.

The single reactor system presented here allows a more straightforward identification of the ideal growth phase for any possible probiotic strain which is required to pass the stomach-intestine passage than if it had to be performed with other systems with a difficult setup.

The study also showed that all tested *Bifidobacterium *strains, except for *B. animalis *subsp. *lactis*, would require protective agents to survive the passage through the stomach-intestine in high numbers. This could be done using an appropriate food matrix or encapsulation of the cells.

## Methods

### Bacterial strains

All bifidobacteria strains were selected from the strain collection of Agroscope Liebefeld-Posieux ALP Research Station Switzerland, isolated by ALP from human sources. *Lactobacillus gasseri *K7 originated from the ZIM Collection of Industrial Microorganisms of University of Ljubljana, Biotechnical Faculty (ZIM 105) [10] and was also deposited in the ALP strain collection. The tested strains and their identification numbers of the ALP strain collection are listed in table 3. All bifidobacteria strains are the property of ALP.

### Media and growth conditions

For pre-cultures, 1 ml frozen conserves of the strains were inoculated in 250 ml Wilkins-Chalgren broth (WC CM0643, Oxoid, Hampshire, UK) supplemented with 9 g l^-1 ^additional lactose-monohydrate (Bifidobacteria) or De Man-Rogosa-Sharpe (MRS; Biolife, Milano, Italy) medium (*Lactobacillus gasseri *K7) [32]. For *L*. gasseri K7, a trial with a 100 ml pre-culture was also performed. All strains, except *Bifidobacterium longum *subsp. *infantis*, were incubated at 37°C for 15 hours under anaerobic conditions. *Bifidobacterium longum *subsp. *infantis *was incubated for 12 h since it was very sensitive to extended incubation periods. The pre-cultures were centrifuged for 15 min at 3500 rpm and the pellets resuspended in 10 ml of phosphate-buffered physiological sodium chloride solution (PBS).

### Determination of cell count

The cell count was determined by 10-fold serial dilution of the culture in physiological saline solution. The two highest dilutions were then plated on MRS agar (Biolife, Milano, Italy) using a spiral plater (IUL Instruments, Barçelona, Spain) and evaluated by an automated colony counter with the corresponding software (IUL Instruments, Barçelona, Spain).

### Screening for acid resistance

For the acid resistance screening the concentrated cell suspension from the pre-culture was pipetted into 20 ml of PBS until an OD_650 _of 1.0 was reached. 4 ml of this cell suspension were then inoculated in 16 ml of citrate-HCl buffer (tri-Na-Citratex2 H_2_O 7.35 g and 250 ml distilled H_2_O, adapted to the corresponding pH with 1 M HCl) at pHs of 2.0, 2.5, 3.0, 3.5 and 4.0. The incubation was done at 37°C and samples were taken every 30 min over 120 min. 1 ml of samples were mixed with 9 ml 0.25 M phosphate buffer at pH 7.0 at the first step of the dilution series. For the acid resistance test in a food matrix, the same amount of pre-culture as used above (adjusted to an OD_650 _of 1.0) was pipetted into 20 ml of UHT skim milk. 4 ml of this cell suspension in milk were inoculated into 16 ml of citrate-HCl buffer. All chemicals were purchased from Merck (Darmstadt, Germany). The data for the screening experiments was visualized in contour plots using the Sigmaplot 11.0 software (Systat Software Inc., Chicago IL, USA).

### Simulation in the bioreactor

All solutions were freshly prepared for each experiment. Simulated stomach solution was made of 50 mg pepsin porcine gastric mucosa (Sigma-Aldrich P7012, Buchs, Switzerland) in 20 ml of 0.1 M HCl. For the simulated pancreatic juice 2 g pancreatin (Sigma-Aldrich P7545) were dissolved in 50 ml of 0.02 M phosphate buffer at a pH of 7.5. Simulated bile salt solution was made of 7.5 g bovine bile (Sigma-Aldrich B3883) made up to 50 ml with distilled H_2_O. The broth for the simulation was either 1 l WC or MRS broth with 29.41 g tri-sodium citratex2 H_2_O. During testing of survival in a food matrix, 500 ml of UHT skim milk were added and the pH adjusted to 3.0 with 5 M HCl shortly before the simulation. 1 l medium was added to the bioreactor (NewMBR Mini, NewMBR, Switzerland), previously sterilized with water (121°C, 20 min), and heated to 37°C. During the stomach simulation, aeration was implemented. The fermentation was controlled and recorded using the integrated process management software Lucullus (Biospectra, Schlieren, Switzerland). The concentrated cell suspension from the pre-culture was pipetted into 40 ml of PBS to an OD_650 _of 1.5. Shortly before the inoculation of 40 ml cell suspension, 20 ml of the simulated stomach solution was added to the medium (1 l) in the bioreactor. The pH was adjusted using 2 M NaOH.

Sixty minutes after the inoculation of the cells, the oxygen was replaced by nitrogen to obtain an anaerobic atmosphere. This was performed by flushing the headspace and making the system air-tight. After attaining a pH of 5.0 (after approx. 1 h fermentation time), 34 ml of the bile salt solution and 50 ml pancreatic juice were inoculated. Samples were taken every 20 minutes during the first hour and then only every 60 minutes. The total simulation time was set to 7 hours with an average stomach pH of 3.0. The time in the stomach was set to one hour, followed by rapid neutralization to 6.3 and a slow increase to 7.5 over the remaining 5 hours and 40 minutes (Figure 1).

## Authors' contributions

RIP conceived and planned the study, evaluated the results and drafted the manuscript. CHK performed the experiments and evaluated the results. VOA revised the manuscript and produced the final version. All authors read and approved the manuscript.
